# Relationship between lexical, reading and spelling skills in bilingual language minority children and their monolingual peers

**DOI:** 10.3389/fpsyg.2023.1121505

**Published:** 2023-08-10

**Authors:** Giulia Vettori, Oriana Incognito, Lucia Bigozzi, Giuliana Pinto

**Affiliations:** Department of Education, Languages, Intercultures, Literatures and Psychology, University of Florence, Florence, Italy

**Keywords:** bilingual language minority children, lexical competence, reading decoding skills, writing orthographic skills, predictivity

## Abstract

This study was conducted on a population of primary school children including bilingual language minority (BLM) children with L2-Italian and a variety of languages as L1 (e.g., Chinese, Albanian, Latin), and Italian-speaking monolingual children. The variety of languages ecologically reflects the nowadays composition of classes in the Italian school system. The aims were to investigate in both linguistic groups: (1) the developmental patterns of lexical, reading and spelling skills; (2) the pattern of predictive relations between lexical, reading and spelling skills. 159 primary school children from Grade 2 to Grade 5 participated in the study: BLM (*n* = 80) and monolingual (*n* = 79) children aged between 7 and 11 years. Each participant completed a vocabulary task (lexical skills), a text reading task (reading accuracy and reading speed) and a text dictation task (orthographic errors). ANOVA statistics showed the comparison of patterns between monolingual and BLM children in lexical, reading, and writing skills. Results show lower performances in lexical, reading and spelling skills in BLM children learning Italian as a second language compared to monolingual peers. Second, partial correlations performed separately for monolinguals and BLM with lexical ability as a control variable, illustrated that all variables correlated with each other in both groups. This result provides the option of performing hierarchical regressions. Finally, hierarchical regression analyses showed that the pattern of predictive relations between lexical, reading and spelling skills is the same across language groups, with the key role of orthographic accuracy as the pivotal process around which reading and lexical skills are built.

## 1. Introduction

One of the primary goals of schooling is to become proficient in literacy processes. Adequate reading and spelling skills are crucial for communication, learning, and school success. Unfortunately, a large proportion of students struggle with reading and writing (Organisation for Economic Co-operation and Development, 2016). This is concerning, because poor reading and spelling skills have negative long-term effects associated with school dropout and unemployment. Data informs that foreigners and first-generation foreign students generally have lower scores in literacy in comparison to natives [for example, National Institute for the Evaluation of the Education and Training System (INVALSI), 2022]. Regarding the population of bilinguals, the debate on the advantages is still open and research results are not always consistent (e.g., Gunnerud et al., 2020; Monnier et al., 2022). For example, evidence are contrasting in regard of bilinguals’ semantic and phonological abilities; a part of results show lower performances of bilinguals that monolinguals (Costa and Caramazza, 1999; Sandoval et al., 2010); while other results did not find any differences between the two language groups (e.g., Friesen et al., 2015). The fragmented picture is strongly linked to the fact that previous researches have been conducted on a wide range of linguistic and metalinguistic performances and tasks, as well as on different populations, such as simultaneous bilinguals (e.g., Farangi and Mehrpour, 2022) or immigrant children (keller et al., 2015)with diverse first and second languages that challenge generalizability of results. Thus, there is the necessity to conduct research that makes explicit the characteristics of the particular type of bilingualism under study, along with the necessity of interpreting results in relation to the specific type of bilingualism under study.

Some recent studies conducted in Italian primary schools have shown that bilingual language minority children underperform in emergent literacy skills such as notational awareness (Incognito et al., 2021); spelling (Bonifacci et al., 2020, 2022; Vettori et al., 2022a,b) and lexical skills compared to monolingual peers. The population of bilingual language minority children represent a very specific type of bilingualism and refer to those children who are exposed to the acquisition of the second language in an intensive and regular way only when they start formal instruction at school, while they continue to use their first language to communicate and interact at home (Hoff et al., 2021). Moreover, bilingual language minority children may face a second language that is very different from their mother tongue, such as in the case of L1-Chinese and L2-Italian which is an alphabetic language with a transparent orthography. In a transparent language almost every sound letter represents only one sound (e.g., Italian, Spanish). A two-year longitudinal study by Pinto et al. (2015) analyzed the relations between reading and writing skills in Italian speaking monolingual children in Grade 1 and Grade 2 of primary school. The results of the cross-lagged analysis showed that early progress in spelling skills later became a resource for reading skills. The study by Desimoni et al. (2012) conducted with Grade 1-to-Grade3 Italian primary school children provided additional support for the key role of spelling which influenced text comprehension and reading speed. In the transparent language, studies identified relations between reading and spelling in samples of children diagnosed as dyslexic in Grade 3 followed longitudinally since kindergarten (Bigozzi et al., 2016a) and Grade 1 in primary school (Bigozzi et al., 2016b). Such studies have been able to identify reading-writing relations in the early phase of acquiring Italian, a transparent writing system, and to contribute to the debate from a cross-linguistic perspective. However, to inform teachers about difficulties that bilingual language minority children could encounter and develop effective interventions to resolve such literacy problems more research is needed, because to date most of the research examines reading and writing relations in monolingual children. Relatively little research exists on bilingual language minority children acquiring a transparent second language. To contribute in filling this gap, this study aimed at investigating the patterns of development and relations between lexical, reading and spelling skills in school-aged monolingual and BLM children with L2-Italian.

### 1.1. Reading, spelling, and their reciprocal relations

Children’s reading and writing skills are supported by overlapping general cognitive domain processes (working memory and executive functions), language skills (lexical and oral narrative skills), and discourse knowledge. Recently, models of writing in literature [for example, “Direct and Indirect Effects Model of Writing” (DIEW)] by Kim and Park (2019) and Kim (2020); the Interactive Dynamic Model (IDM) by Kim (2020) and Kim and Graham (2022) show a complex pattern of relations between reading and writing domains. From a developmental point of view, children learn to read far earlier than they learn to write, thus reading skills may be pivotal for writing development. However, research on the relationship between reading and writing domains has shown that bidirectional models are the most powerful with respect to reading-to-writing models or writing-to-reading models (Shanahan and Lomax, 1988). Recent research has focused on the existence of articulated patterns of relationships between reading and writing when word, sentence, and text levels are implied. For example, Kim et al. (2018) conducted a longitudinal study comprising students in Grades 3 to 6 in the US, and the results showed that bivariate correlations between reading and spelling were strong across all grades at the word level, even if reading–writing relations (for instance, reading comprehension and written composition) were weak at the discourse level. Whereas, Ahmed et al. (2014) demonstrated that a reading-to-writing model for grades 1 to 4 better described the data for the word and text levels, but a bidirectional model best fit the data at the sentence level. Findings from these studies suggest that reading and writing are related, but the patterns of the relationship need to be investigated across different populations (monolingual and bilingual language minority children) and languages with different levels of orthography depth. Lexical skills, including depth and quantity of words known, are necessary for reading and writing. As children become proficient in lexical skills, they can devote their cognitive resources to higher-order cognitive processes. Previous studies have shown that Italian–English bilinguals (Roch et al., 2016) and Chinese–Italian bilingual language-minority children (Vettori et al., 2022a) have lower lexical skills in L2 than in L1 in comparison to their monolingual peers in primary school, and this disadvantage has a negative repercussion on their text writing outputs. Regarding the impact of the linguistic condition on literacy, to date, there is an open debate about whether, and to what extent, limited input of L2-societal language at home affects reading-writing development in bilingual language-minority children. Differences between L1 and L2 writing processes were also demonstrated by studies in which writing processes in the first language (L1) and a second language (L2) were compared (Tillema et al., 2013). However, little is known about the pattern of development and relations between lexical, reading and spelling skills in primary school BLM children learning a transparent second language, such as Italian. Regarding the effects of the characteristics of the language system on reading and spelling relations, recent research stresses the need to consider the role of the specific orthography. Results from opaque orthographies such as English, in which the same letter can represent different phonemes, cannot be generalized to more transparent orthographies, such as Spanish, Finnish, Greek and Italian. In fact, the biunivocal correspondence between sounds in a highly transparent language influences the types of reading and spelling errors made by children and influences reading and writing trajectories. Children speaking a language whereby the transcription is almost transparent are expected to automatise reading and spelling skills within the first two years of schooling; the spelling errors that may persist are homophone errors rather than non-homophone errors. Previous longitudinal studies in monolinguals (e.g., Pinto et al., 2015) suggesting that early progress in spelling skills became a resource for later reading skills ask for further investigation on the specific population of bilingual language-minority children that may be configured as “at-risk” for reaching adequate reading and writing acquisitions in the early school years. We know from literature that the condition of bilingual language-minority (BLM) children with L2-Italian can be associated with lower level of notational awareness and phonological awareness which are key precursors of reading and writing, because of the limited possibility to practice L2-Italian within daily home literacy practices.

### 1.2. Aim and hypothesis

To deepen our knowledge about the relationship between reading, writing, and lexical skills of bilingual language-minority children compared to that of their monolingual peers, this study examined a group of bilingual-language minority children characterized by high L1-linguistic heterogeneity [for example, Chinese, Albanian and Romans] and Italian as L2, a language comprising a writing system that relies on great transparency and in their monolingual peers in primary school (grade 2 to 5).

Specifically, we first examined the developmental patterns of lexical, reading, and spelling skills by comparing the performance of the two language groups. Based on previous research, it was expected that bilingual language-minority children with Italian as a second language would show significantly lower lexical, reading, and spelling skills than their monolingual peers due to the limited L1 input at home.

Second, we investigated the pattern of predictive relations between reading and spelling skills in the two language groups with lexical skills as a control variable, given its strong relation with reading and writing (Kim et al., 2014). Based on the studies in the literature that highlight the shared set of skills and knowledge between reading and writing domains, it was expected that those skills were related in both monolingual and BLM children. However, we cannot anticipate whether the two language groups have a similar pattern of the relationship or if the pattern varies because of the specificity of the orthography under study.

## 2. Method

### 2.1. Participants

159 primary school children comprising BLM (*n* = 80) and monolinguals (*n* = 79) aged between 7 and 11 years (*M*-age = 8.58, *SD* = 1.15; 41% girls and 59% boys) participated in the study. All children attended primary school in a city in the center of Italy from Grade 2 to Grade 5. 22% of participants attended the 2nd grade, 25% 3rd grade, 28% 4th grade and 25% 5th grade. Bilingual language minority children had exposure to an L1 other from Italian (L2) within the family context, as assessed *via* a questionnaire completed by parents. All the bilingual language minority children were born in Italy. To ensure bilingual children’s levels of language proficiency in L2 (i.e., Italian) it was ascertained that all the children were schooled in Italian. To ensure that any other factors may interfere with results on second language acquisition, children with any known special educational needs or impairments/disorders were excluded from the analysis. The school authorities, parents, and children consented to the study. In Italy, first-grade teachers focus primarily on the spelling component of writing, whereas second-grade teachers focus on the textual properties of writing because second-graders are expected to finalize the acquisition of orthography (Ministry of Education, Universities and Research, 2012).

### 2.2. Measures

#### 2.2.1. Lexical skills

Children’s lexical skills were assessed through Multidimensional Vocabulary Tasks (Boschi et al., 1989, 1996) designed for the Italian language. The test evaluates the ability to define words by implementing the cognitive-linguistic processes of categorization based on perceptual and functional attributes, and the ability to construct synonyms and antonyms. The test also evaluates the ability to define the contextually correct meaning of polysemic words that are frequently used in Italian, such as bello (beautiful), buono (good) and grande (big), which have different meanings depending on the phrasal context in which they are used. Following the procedure reported in the test manual, the children were asked to read a short written text (50–100 words) and answer 20 multiple-choice questions regarding the meaning of some of the words. Before the test began, a familiar reading of the task was conducted, and the children were allotted an appropriate test based on their school years. Based on the test manual, each child received a final lexical correctness score, with scores ranging up to 20. Regarding our sample, Cronbach’s alpha was 0.78.

#### 2.2.2. Reading skills

The MT reading test (Cornoldi et al., 1998) is a standardized test with strong psychometric properties commonly used in the Italian educational system. The test was administered individually by the researcher, who asked each child to read the text aloud as best as he/she could, while the researcher noted the reading time and errors. This test produces two scores for reading accuracy and speed. Reading accuracy takes into account the number of errors made by the student while reading aloud, that is, mispronunciations, omitted words, or added syllables, as well as pauses longer than 5 s. Each type of error was counted only once during the test. Reading rapidity refers to the ratio of time that the student takes to read the paragraph (in seconds) and the total number of syllables read. Hence, the slower the children read, the higher the score. For psychometric parameters, please refer to the manual (Cornoldi et al., 1998).

#### 2.2.3. Spelling skills

Standardized dictation was used to measure children’s spelling skills. Paper-and-pencil text dictation was performed individually by children in a collective session in the classroom during school time. The dictation task was taken from the “Battery for the Evaluation of Writing and Orthographic Competence in Primary School” (Tressoldi and Cornoldi, 2000) standardized for the Italian population. Text dictation allows one to analyze children’s spelling skills within an ecological setting provided by the semantic context. The appropriate dictation text was used according to the grade. The children listened to the recorded text and each child was required to write the text. To measure children’s spelling skills using dictation, the orthographic errors were identified based on the classification of the orthographic errors by Pinto et al. (2012) that distinguishes between homophone and non-homophone errors, thus covering the entire variability of orthographic errors. In fact, homophone errors occur when the pronunciation of the target word is preserved despite the spelling error [for example, “anno” (year) instead of “hanno” (they have)]. Non-homophone errors occur when the pronunciation of the target word is changed due to a spelling error (“mecrato” instead of “mercato”). Spelling skills were calculated according to the number of homophone and non-homophone orthographic errors; these were counted according to the number of times the errors occurred. In the manual of the instrument, the test–retest reliability regarding errors ranged from 0.57 to 0.84.

### 2.3. Data analysis

Before analyzing the data, the presence of univariate outliers was checked (refer to Tabachnick and Fidell’s recommendation, Tabachnick and Fidell, 2013). One univariate outlier was found and eliminated.

As a preliminary step, ANOVA was used to examine the evolution of the pattern of reading and writing, as well as lexical skills in the BLM and monolingual groups. In the case of non-homogeneity of variances, ANOVA (with robust methods) was used (Welch Test).

In the next phase, to assess the relationship between two reading skills (rapidity and accuracy) and one writing skill (accuracy), a preliminary partial correlation analysis was separately computed for BLM and monolingual peers, controlling lexical competence.

Following Musca et al. (2011) and Maas and Hox (2005) suggestions, three hierarchical regression analysis models were used to verify the predictive value of the relationship between reading and writing skills by controlling for lexical competence, in both BLM and monolingual peers. This is generally assessed by testing the change in R-squared from one model to the next. If, after the inclusion of predictors at a given step, the change in the R-squared score was significantly greater than zero, we inferred that the predictors included in that step offered incremental predictive power. The R-squared change (increment) from Model 1 to Model 2 was computed as ΔR^2^ = Model 2 R^2^ - Model 1 R^2^. The R-squared change (increment) from Model 2 to Model 3 was computed as ΔR^2^ = Model 3 R^2^ - Model 2 R^2^, and so on (Darlington & Hayes, 2017). In the first analysis, writing skills (accuracy) were the dependent variable, and reading skills (both rapidity and accuracy) were the independent variable. In the second and third analyses, rapidity and accuracy reading skills were the dependent variable, and writing skills were the independent variable.

## 3. Results

Preliminary descriptive statistics and comparative analyses between monolinguals and BLM (utilizing One-Way ANOVA and ANOVA with the robust method - Welch’s Test) of the main variables are shown in Table 1. Statistically significant differences were found between monolingual and BLM children in reading accuracy, writing accuracy, and lexical competence. Specifically, BLM children exhibited a significantly higher number of errors than monolingual children in reading and writing skills. Regarding lexical competence, BLM children performed significantly worse than their monolingual peers.

**Table 1:** Descriptive statistics (mean, SD, minimum and maximum), test homogeneity statistics and results of ANOVA differences between Monolinguals and BLM.

		Mean	SD	Minimum	Maximum	Levene’s statistic^1^	Df	*F*
Reading rapidity	Monolinguals	0.38	0.22	0.17	1.23	0.27	1, 97	3.59
	BLM	0.46	0.23	0.21	1.12			
Reading accuracy	Monolinguals	3.38	2.41	0	12	12.97***	1, 100	12.19***
	BLM	5.94	4.58	0	19			
Writing accuracy	Monolinguals	12.07	12.12	0	56	4.35*	1, 145	9.68**
	BLM	18.92	14.43	0	62			
Lexical competence	Monolinguals	10.46	4.86	−1	17	0.07	1, 136	27.45***
	BLM	6.21	4.66	−3	17			

*p < 0.05; **p < 0.01; ***p < 0.001; ^1^ in the case of significance: *F* statistics were computed with Welch test.

Partial correlation analyses were conducted to determine the relationships between the variables by controlling lexical competence. Writing skill scores were associated with both reading rapidity and reading accuracy. The results are presented in Table 2.

**Table 2:** Partial correlation for both groups: monolingual and BLM children.

	Control variable		Reading rapidity	Reading accuracy	Writing accuracy
Monolingual children	Lexical competence	Reading rapidity		0.39*	0.70***
		Reading accuracy			0.33*
		Writing accuracy			
BLM children	Lexical competence	Reading rapidity		0.55***	0.71***
		Reading accuracy			0.54***
		Writing accuracy			

*p < 0.05; ***p < 0.001.

The first hierarchical regression analysis was performed in three steps to determine whether performance in reading rapidity and accuracy skills improved participants’ prediction of their writing accuracy beyond those provided by lexical competence. These predictors were used in the equation because of their statistically significant correlations with writing skills. Table 3 illustrates the standardized regression coefficients (β), R^2^, and change R^2^ (ΔR2) for the monolingual and BLM children.

**Table 3:** First hierarchical regression analysis for monolingual and BLM children (dependent variable: writing accuracy skill).

		β	R^2^	Δ R^2^
Monolingual children				
Step 1	Lexical competence	−0.36*	0.13*	
Step 2	Lexical competence	−0.27	0.22*	0.09
	Reading accuracy	0.32*		
Step 3	Lexical competence	−0.10	0.56***	0.34
	Reading accuracy	0.06		
	Reading rapidity	0.68***		
BLM children				
Step 1	Lexical competence	−0.32	0.10	
Step 2	Lexical competence	−0.18	0.36***	0.26
	Reading accuracy	0.53***		
Step 3	Lexical Competence	−0.10	0.58***	0.22
	Reading accuracy	0.20		
	Reading rapidity	0.59***		

*p < 0.05; ***p < 0.001.

In Model 1, for monolingual children, lexical competence accounted for significant variations in writing accuracy skills, R-square = 0.13, *F* (1, 38) = 5.56, *p* = 0.024). In Model 2, lexical competence and reading accuracy skills accounted for significant variations in writing accuracy skills [R-square = 0.32, *F* (1, 37) = 4.45, *p* = 0.042]. The change in R-squared from Model 1 to Model 2 was 0.09, reflecting a significant increase in the explained variation. In Model 3, the predictors accounted for significant variations in writing accuracy skills, R-squared = 0.56, *F* (1,36) = 27.80, *p* < 0.001). The change in R-squared from Model 2 to Model 3 was 0.34, which reflects a significant increase in the explained variation. These results show that the only predictor of writing accuracy in monolingual children is reading rapidity.

In Model 1, for BLM children, lexical competence did not account for significant variations in writing accuracy skills, R-squared = 0.10, *F* (1, 36) = 4.02, *p* = 0.053). In Model 2, lexical competence and reading accuracy skills accounted for significant variations in writing accuracy skills (R-squared = 0.36, *F* (1, 35) = 14.11, *p* < 0.001). The change in R-squared from Model 1 to Model 2 was 0.26, reflecting a significant increase in the explained variation. In Model 3, the predictors accounted for significant variations in writing accuracy skills, R-squared = 0.58, *F* (1,34) = 18.18, *p* < 0.001). The change in R-squared from Model 2 to Model 3 was 0.22, which reflects a significant increase in the explained variation. In addition, the results show that the only predictor of writing accuracy skills in monolingual children is reading rapidity.

The second and third hierarchical regression analyses were performed in two steps to determine whether performance in writing accuracy skills improved participants’ prediction of their reading rapidity skills (Table 4) and reading accuracy skills (Table 5), beyond those provided by lexical competence. These predictors were used in the equation because of their statistically significant correlations with writing skills. Tables 4, 5 illustrate the standardized regression coefficients (β), R^2^, and change R^2^ (ΔR2) for monolingual and BLM children, respectively.

**Table 4:** Second hierarchical regression analysis for monolingual and BLM children (dependent variable: reading rapidity skill).

		Β	R^2^	Δ R^2^
Monolingual children				
Step 1	Lexical competence	−0.44**	0.19**	
Step 2	Lexical competence	−0.11	0.65***	0.46
	Writing accuracy	0.75***		
BLM children				
Step 1	Lexical competence	−0.27	0.07	
Step 2	Lexical competence	−0.04	0.57***	0.50
	Writing accuracy	0.74***		

**p < 0.01; ***p < 0.001.

**Table 5:** Third hierarchical regression analysis for monolingual and BLM children (dependent variable: reading accuracy skill).

		Β	R^2^	Δ R^2^
Monolingual children				
Step 1	Lexical competence	−0.31*	0.09*	
Step 2	Lexical competence	−0.19	0.18*	0.09
	Writing accuracy	0.31*		
BLM children				
Step 1	Lexical competence	−0.32*	0.10*	
Step 2	Lexical competence	−0.12	0.34***	0.24
	Writing accuracy	0.52***		

*p < 0.05; ***p < 0.001.

In the second hierarchical regression, for monolingual children in Model 1, lexical competence accounted for significant variations in reading rapidity skills (R-squared = 0.19, *F* (1, 45) = 10.68, *p* = 0.002). In Model 2, lexical competence and writing accuracy skills accounted for significant variations in reading rapidity skills (R-squared = 0.65, *F* (1, 44) = 56.36, *p* < 0.001). The change in R-squared from Model 1 to Model 2 was 0.46, reflecting a significant increase in the explained variation. These results show that writing accuracy is the only predictor of reading rapidity in monolingual children.

In Model 1, lexical competence did not account for significant variations in reading rapidity skills, R-squared = 0.07, *F* (1, 38) = 3.05, *p* = 0.089). In Model 2, lexical competence and writing accuracy skills accounted for significant variations in reading rapidity skills (R-squared = 0.57, *F* (1, 37) = 43.00, *p* < 0.001). The change in R-squared from Model 1 to Model 2 was 0.50, reflecting a significant increase in the explained variation. These results show that writing accuracy is the only predictor of reading rapidity in monolingual children.

In the third hierarchical regression, for monolingual children, lexical competence accounted for significant variations in reading accuracy skills in Model 1 [R-squared = 0.09, *F* (1, 43) = 4.44, *p* = 0.041]. In Model 2, lexical competence and writing accuracy skills accounted for significant variations in reading accuracy skills [R-squared = 0.18, *F* (1, 42) = 4.43, *p* = 0.041]. The change in R-squared from Model 1 to Model 2 was 0.09, reflecting a significant increase in the explained variation. These results show that writing accuracy is the only predictor of reading accuracy in monolingual children.

In Model 1, for BLM children, lexical competence accounted for significant variations in reading accuracy skills, R-squared = 0.10, *F* (1, 42) = 4.78, *p* = 0.034). In Model 2, lexical competence and writing accuracy skills accounted for significant variations in reading accuracy skills [R-squared = 0.34, *F* (1, 41) = 14.47, *p* < 0.001]. The change in R-squared from Model 1 to Model 2 was 0.24, reflecting a significant increase in the explained variation. These results show that writing accuracy is the only predictor of reading accuracy in monolingual children.

However, in both monolinguals and BLM, the results illustrated that the total variance explained by the model with the dependent variable, that reading rapidity was greater than the variance explained by the reading accuracy variable.

**Figure 1:**
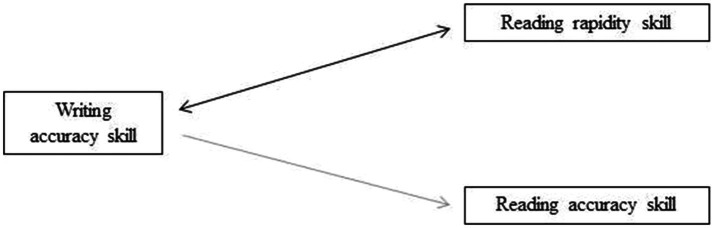
Graphical representation of results on the predictive value of variables.

Finally, Figure 1 presents a summary of the predictive values among the variables. Specifically, demonstrating that writing accuracy and reading rapidity predict one another, whereas writing accuracy predicts reading accuracy.

## 4. Discussion

Two overarching questions guided this study: (a) What is the pattern of development of lexical, reading and spelling skills in primary school monolinguals and BLM children? (b) What is the pattern of relations between lexical, reading and spelling skills in primary school monolinguals and BLM children? We focused on primary school children, from grades 2 to 5, when children are expected to acquire reading and spelling skills. Few studies have examined reading-writing reciprocal relations, by controlling lexical skills in bilingual language minority children who experience learning to read and write in Italian (a transparent language system) as a second language. The importance of lexical skills for learning to read and write is documented by the Dual Route Cascaded Model (Coltheart et al., 2001) in literature. The use of the lexical route is highest in experienced writers and readers (Perfetti, 1989, 1997; Booth et al., 2000). Also, the relation between lexical skills with reading and writing is well-documented by research results obtained in opaque (Kim et al., 2014) and transparent orthographies (Bigozzi and Biggeri, 2000).

We found different patterns of development of lexical, reading and spelling skills when comparing performances between monolinguals and BLM children. Monolingual children outperformed their BLM peers in lexical, reading and spelling skills. In fact, the comparison of performances in the lexical task showed that BLM children had a lower vocabulary knowledge depth and ability to define words through cognitive-linguistic processes in L2-Italian (for example, errors in constructing synonyms and antonyms) than monolinguals. Furthermore, BLM children made more errors such as mispronounced or omitted words, or added syllables in a text-reading task in L2-Italian compared to their monolinguals peers, while BLMs’ reading rapidity skills are at the same level as monolinguals. In addition, BLM children made more spelling errors, such as inversion and substitution of letters and disregarded orthographic rules, in a text dictation task in L2-Italian than monolinguals. The results of BLMs’ poor performances that emerged in lexical, reading and spelling skills are partially in accordance with the literature. Specifically, our results aligned with previous studies investigating bilingual language-minority children with L1-Chinese and L2-Italian whose results demonstrated difficulties in literacy skills during different periods of development (for example, Bonifacci and Tobia, 2016; Vettori et al. 2022a). While there may be several explanations, we speculate that these results are attributed, at least partially, to the fact that BLM children grow up with less L1-Italian language input and opportunities to practice the L1-Italian language beyond school. Moreover, while all monolingual children participating in our research attended pre-school extensively, this experience was rare (and brief) in bilingual children. A study by Incognito et al. (2021) demonstrated that notational knowledge, defined as preschoolers’ knowledge about the correspondence between phoneme and grapheme, was lower in bilingual language-minority (BLM) preschoolers than in their monolingual peers. This could be linked to BLM difficulties in spelling and reading accuracy in primary schools. The writing system constitutes highly conventional and arbitrary material, the formalized learning of which occurs in elementary school, but whose roots are already present in kindergarten, in the period of emergent literacy. Numerous studies have documented the strong predictive relevance of emergent literacy skills for the acquisition and mastery of conventional encoding and decoding skills (Pinto et al., 2015; Bigozzi et al., 2016a).

Interestingly, our results showed that the level of reading rapidity was quite similar between the two language groups (monolingual and BLM peers). In other words, monolingual and BLM children in primary school take the time to read a narrative passage despite their differences in reading accuracy. It appears that reading accuracy and rapidity are independent constructs, even if they are related. Regarding the non-difference in the speed of reading a narrative, again keeping lexical competence in mind, we can hypothesize that the specific type of text contributes to this result. Narratives are widely distributed in all languages, familiar to children, and highly practiced in all family experiences. This may provide all children with easy anchoring to the structural properties of the text, and easy use of the contextual knowledge contained in the story; thereby accelerating the top-down processes of reading, particularly the formulation of hypotheses about words and their completion, which supports reading speed.

Regarding the relationship between reading and spelling skills by controlling lexical skills, the results of the partial correlations showed that the variables were related in both language groups; this result was confirmed by regression models. Regression models revealed that lexical skills supported spelling and reading skills in the Italian language in both language conditions (i.e., monolingual or BLM children). However, once reading or spelling variables were added to the regression models, the predictive contribution of lexical skills disappeared in favor of the reciprocal predictive role of reading and spelling skills. The strong association between reading and spelling skills is consistent with the fact that reading and spelling rely on highly similar skills, such as phonological awareness, orthographic awareness, morphological awareness (Berninger et al., 1998; Kim et al., 2013), oral language skills, lexical-level literacy skills, higher-order cognitive skills, and self-regulatory processes (for example, Berninger and Abbott, 2010; Kim & Graham, 2022). The present study revealed that literacy skills such as reading and spelling, which are strongly correlated to formalized schooling, form stronger associations with respect to lexical skills, which are connected to the oral language domain and cognitive-linguistic processes. The results concerning the bidirectional relationship between reading and spelling skills are congruent with earlier studies of monolingual primary school children conducted using a variety of languages (for example, Ahmed et al., 2014; Pinto et al., 2015), and allow us to extend our knowledge of how encoding and decoding, and in particular accuracy and speed, interconnect in the course of formalized literacy to bilingual children, acquiring Italian as written language. BLM children acquiring Italian language as L2 show the same pattern of reciprocal relations between reading and spelling skills. The results that emerge demonstrate how in formalized literacy, in a largely spelling-transparent language, reading and writing rely on each other in an interconnected manner, which involves two components of the process: accuracy and rapidity in encoding and decoding words, probably due to the correspondence between sound signs in the Italian language. This interconnectedness, which is significant since the beginning of formalized literacy, becomes more intense as the number of participating children progress through their schooling years. The more that children master reading and spelling skills, the more the two domains (reading and spelling) become interconnected. The results offer an additional, important piece of information: within the overall pattern of interactions between reading and writing, writing accuracy makes the greatest contribution to literacy advancement children utilize their expertise to review and check the correctness of what they have written to improve their speed in writing and to correctly attribute sound value to graphemes, as well as to increase their deciphering speed. It is important to improve our understanding of which are the best practices of teaching to read and write for professional development programs (Rietdijk et al., 2018). From a practical point of view, our findings suggest that for monolinguals and children from homes, whose family speak different languages compared to the societal one, there is a need to promote the transfer of skills between reading and writing, to support literacy development, instead of intervening on each of them sequentially or by repeating the task.

## 5. Limitations and future research

The present research has several limitations. First, the cross-sectional nature of this study needs to be expanded by future longitudinal studies to better inform reading and writing trajectories in different language groups. Second, it could be useful to measure reading and writing abilities by adopting various measures beyond the word level, such as sentence and text levels, to verify the stability of the patterns of relationships that emerged. Finally, a more detailed examination of children’s home literacy environment and practices could help clarify the differences in performance between monolingual and BLM children detected in this sample. Therefore, this kind of research could benefit from examining the data from a cross-linguistic perspective.

## Data availability statement

The raw data supporting the conclusions of this article will be made available by the authors, without undue reservation.

## Ethics statement

The studies involving human participants were reviewed and approved by University of Florence. Written informed consent to participate in this study was provided by the participants' legal guardian/next of kin.

## Author contributions

All authors listed have made a substantial, direct, and intellectual contribution to the work and approved it for publication.

## Conflict of interest

The authors declare that the research was conducted in the absence of any commercial or financial relationships that could be construed as a potential conflict of interest.

## Publisher’s note

All claims expressed in this article are solely those of the authors and do not necessarily represent those of their affiliated organizations, or those of the publisher, the editors and the reviewers. Any product that may be evaluated in this article, or claim that may be made by its manufacturer, is not guaranteed or endorsed by the publisher.
